# Job security among healthcare workers in Guangdong, China

**DOI:** 10.3389/fpubh.2023.1096825

**Published:** 2023-03-01

**Authors:** Qingxia He, Luís M. Dias Martins, Shibin Wang, Qishan Zhan, Xiao Yu, Zhiqiong Ba, Wangjiu Li, Huigen Huang

**Affiliations:** ^1^Center of Guangdong Mental Health, Guangdong Provincial People's Hospital (Guangdong Academy of Medical Sciences), Southern Medical University, Guangzhou, China; ^2^BRU-Business Research Unit ISCTE-IUL (Institute University of Lisbon), Lisbon, Portugal; ^3^Nursing Department, Guangdong Provincial People's Hospital (Guangdong Academy of Medical Sciences), Southern Medical University, Guangzhou, China; ^4^Nursing Department, Guangzhou Kangda Vocational Technical College, Guangzhou, China

**Keywords:** healthcare workers, job security, current situation, influencing factors, a cross-sectional study

## Abstract

**Objective:**

The objective of this study was to explore the sense of job security and its influencing factors among healthcare workers in Guangdong, China.

**Methods:**

This cross-sectional study used stratified random sampling to enroll healthcare workers employed by hospitals across Guangdong province between September 2020 and October 2020.

**Results:**

A total of 4,173 questionnaires were distributed, and 4,076 were returned for an effective recovery rate of 97.68%. The overall score for the sense of security was 64.85 ± 20.09, and the item means score was 2.95 ± 0.91. Multiple-linear regression analysis showed that work experience (years), education level, job position, specialty unit, employment type, marital status, job satisfaction, WPV frequency, daily sleep duration, weekly overtime hours, average monthly earnings (RMB), hospital level, and region were significantly associated with senses of poor security among healthcare workers (all *P* < 0.05).

**Conclusions:**

Hospital workers in Guangdong reported relatively low levels of job security. Levels of job security were significantly associated with multiple factors which could be addressed by hospital practices to improve the sense of job security among healthcare workers.

## 1. Introduction

Safety is the most basic physiological need of human beings (1), and lack of job security not only affects job satisfaction, but also leads to the lack of job safety and quality (2). Poor doctor-patient relationships are a significant concern for healthcare workers in China (3, 4). Approximately two-thirds of physicians have reported disputes or doctor-patient conflicts which threatened their physical safety (5). A recent article published in the Lancet examining the safety of healthcare workers in China concluded that medical workers in China have a very low sense of security (6).

The recent coronavirus pandemic only made safety concerns among hospital workers in China more apparent (7). A Chinese report on workplace violence in hospitals showed that 100% of nurses expressed lack of job security (8). An American study reports that 34.4% of medical workers experienced physical violence within 12 months, and 13.5% suffered physical attacks (9). Among those who suffered verbal or physical violence, 9.4% did not want to continue working in the medical industry.

As the above data indicate, job security is an important factor which influences the retention of nurses (10). Yan et al. (11) found that there is a relationship between job insecurity, quality of life and turnover intention whereby reduced job insecurity was associated with a lower quality of life and higher turnover intention. A cross-sectional survey which included 403 medical staff members as research objects found that job insecurity was an important predictor of job burnout (12). Similarly, a Chinese study that included 453 nurses found that occupational stress was inversely correlated with psychological security (13). Moreover, over two-thirds of doctors in China report symptoms of burnout in a recent systematic review (14).

Workplace Violence (WPV) is defined by the International Labor Organization (ILO) as the assault of threat of assault to a staff member in a work-related environment that threatens their safety, wellbeing or health (15, 16). The domestic incidence rate of WPV among medical workers in China is 61.9% (17, 18). Further, 68.6% of the healthcare workers in pediatrics in China have experienced WPV at least once over the course of a year (19). Related studies have shown that WPV harms individual mental health and job performance (20) and that anxiety among healthcare workers increased significantly after experiencing WPV (21, 22).

Pienaar's Job Insecurity Scale (JIS-8) is commonly used abroad, and includes two dimensions: emotional job insecurity and cognitive job insecurity (23). However, the reliability and validity of Pienaar's job insecurity scale (JIS-8) has not been verified among workers in China or among healthcare workers. Cong et al. (24) compiled a job security scale for workers in China that includes the dimensions of sense of control and interpersonal security. However, this scale does not take into consideration the professional characteristics of medical staff.

In order to better study job security among healthcare workers in China, a scale was adopted in this study, which was compiled through qualitative and quantitative research on the experience of safety by medical staff (25). This scale better reflects the professional characteristics of the medical staff as compared to currently available surveys. Using this scale, this study examined perceptions of job security among healthcare workers in hospitals of Guangdong province and used these data to identify factors which may influence job security in this population.

## 2. Methods

### 2.1. Study design and populations

This cross-sectional study used stratified random sampling to enroll healthcare workers from hospitals in Guangdong Province between September 2020 and October 2020. This study was conducted stratified random sampling on three groups of hospitals, primary, secondary and tertiary, as classified by the Chinese government. The June 2018 list of general hospitals published by the Commission of Health and Family Planning was used to obtain the names and contact information for 204 primary, 224 secondary, and 76 tertiary hospitals in Guangdong. Hospitals from each of the four regions were included and the sampling ratio of primary hospitals: secondary hospitals: tertiary hospitals was determined to be about 2:1:1. According to health statistics published by the Guangdong Health Commission in 2019, there were 292,128 licensed physicians, 356,784 registered nurses, 43,374 pharmacists and 37,175 technicians working in hospitals in Guangdong in 2019. The sampling ratio of doctors: nurses: pharmacists: technicians, was about 4:8:1:1.

The inclusion criteria were as follows: (1) Age over 18 years; (2) Medical personnel working in clinical posts (doctors, nurses, pharmacists and technicians); (3) Without cognitive dysfunction; (4) Voluntary participation in the survey. Interns, trainees, and employees studying abroad or who were otherwise absent during the study period were excluded. This study was approved by the Guangdong Provincial People's Hospital Ethic Committee (2019236H).

### 2.2. Instruments and procedure

Staff were administered a questionnaire that included items on gender, age (years), work experience (years), education level, job position, specialty unit, professional title, employment type, marital status, job satisfaction, WPV frequency, daily sleep duration, weekly overtime hour, average monthly earnings (RMB), hospital level and region within the province.

The sense of job security scale (25) included 22 items across five dimensions: patients, self, organizational management, social support and environment. A Likert 5-level scoring method was used, and each item was given a score on a discrete scale from 1 to 5. A score of 1 was given if the participant very strongly agreed with that item and a score of 5 was given if the participant very strongly disagreed with that item. The total score ranged from 22 to 110, with score ≥ 44 indicating a high level of job security, while score < 44 indicating a low level of job security. The total Cronbach's alpha coefficient was 0.939, the split-half reliability was 0.96, and the test-retest reliability was 0.967, indicating good internal consistency.

The questionnaire service (https://www.wenjuan.com) was used to administer each survey. Before beginning the survey, each participant was introduced to the software interface and the purpose of this survey and gave written informed consent. In order to avoid repeat submissions, a single account, device and IP address can only be used once to take the questionnaire. The questionnaire service was used to export the data, and the data was manually screened and reviewed by two independent evaluators.

### 2.3. Statistical analysis

According to Kendall's empirical estimation method, the sample size N should be 5–10 times of the number of independent variables (26). The number of independent variables in the subject was 22, indicating that the maximum required sample size according to this method is 220. Reasonably, up to 20% of the questionnaires could be invalid, so a sample size of at least 275 was used.

SPSS version 23.0 (IBM, Armonk, NY, USA) was used for statistical analysis. Continuous variables were described as mean ± SD or median values with interquartile range depending on normality of the variables. Categorical variables were presented as percentages. Comparisons for continuous data were performed using Student t-test or Mann-Whitney U test. Categorical variables were compared using the chi-square test or Fisher exact test. A Multiple linear regression model was used to analyze the factors influencing healthcare workers security. All analyses were two-sided, and *P*-values < 0.05 were considered statistically significant.

## 3. Results

A total of 29 hospitals participated in this study, including 14 tertiary hospitals, 8 secondary hospitals, and 7 primary hospitals. A total of 4,173 questionnaires were distributed, among which 97 were invalid and excluded (55 working for < 1 year, 35 administrative and logistics staff, and 7 refused to participate), and 4,076 were finally recovered for an effective recovery rate of 97.68%. Among the 4,076 doctors, nurses, pharmacists and technicians that completed the survey, 231 (5.7%) worked in primary hospitals, 1,462 (35.9%) in secondary hospitals and 2,383 (58.5%) in tertiary hospitals (Table 1). A total of 2,426 (59.5%) staff worked in the Pearl River Delta, 377 (9.2%) in eastern Guangdong, 703 (17.2%) in western Guangdong and 570 (14.0%) in northern Guangdong. There were 1,484 males (36.4%) and 2,592 females (63.6%), with a mean age of 32.46 ± 9.06 years old. The average duration of employment was 10.75 ± 8.4 years. There were 2,238 (54.9%) nurses, 1,019 (25.0%) doctors, 438 (10.7%) pharmacists, and 381 (9.3%) technicians.

**Table 1:** General information on medical personnel (*n* = 4076).

**Item**		**Subjects (*n*, %)**	**Scores (mean ±SD)**	***P***
Gender	Male	1,484 (36.4)	63.99 ± 19.89	0.039
	Female	2,592 (63.6)	65.34 ± 20.19	
Age (years)	≤ 25	984 (24.1)	64.48 ± 20.62	0.180
	26-	1,831 (44.9)	64.61 ± 20.08	
	36-	871 (21.4)	64.84 ± 19.92	
	≥46	390 (9.6)	66.96 ± 19.07	
Work experience (years)	≤ 5	1,376 (33.8)	64.58 ± 20.05	0.004
	6-	1,172 (28.8)	64.21 ± 19.92	
	11-	882 (21.6)	64.21 ± 20.71	
	≥21	646 (15.8)	67.48 ± 19.45	
Education level	Junior college or below	1781 (43.7)	63.86 ± 20.34	< 0.001
	Bachelor's degree	1,363 (33.4)	64.65 ± 19.57	
	Master's degree or above	943 (22.9)	67.04 ± 20.21	
Job position	Doctor	1,019 (25.0)	64.87 ± 19.45	< 0.001
	Nurse	2,238 (54.9)	62.83 ± 21.18	
	Pharmacist	438 (10.7)	70.45 ± 16.28	
	Technologist	381 (9.3)	70.23 ± 16.68	
Specialty unit	Intensive Care Unit	228 (5.6)	61.22 ± 18.15	< 0.001
	Medical	1,087 (26.7)	63.52 ± 20.61	
	Surgical	698 (17.1)	63.53 ± 20.91	
	Maternity	221 (5.4)	61.16 ± 19.98	
	Pediatric	136 (3.3)	61.15 ± 18.58	
	Outpatient/emergency department	448 (11.0)	65.13 ± 21.95	
	Assistant department	1,098 (26.9)	69.14 ± 17.71	
	Other	160 (3.9)	62.89 ± 22.15	
Professional title	Primary	2,465 (60.5)	64.46 ± 19.82	0.060
	Middle	1,214 (29.8)	64.94 ± 20.83	
	Advanced	397 (9.7)	67.03 ± 19.37	
Employment type	Contract employee	2,369 (58.1)	62.81 ± 19.94	< 0.001
	Permanent employee	1,632 (40.0)	67.81 ± 19.83	
	Personnel agency employee	75 (1.8)	64.95 ± 22.60	
Marital status	Married	2,655 (65.1)	67.63 ± 18.40	< 0.001
	Single	1,339 (32.9)	59.78 ± 21.67	
	Divorced/separated	82 (2.0)	57.74 ± 27.04	
Job satisfaction	Yes	1,198 (29.4)	73.35 ± 20.97	< 0.001
	No	2,878 (70.6)	61.31 ± 18.60	
WPV frequency	No	1,865 (45.8)	69.62 ± 21.20	< 0.001
	≤ 3 times	1,710 (42.0)	61.45 ± 18.52	
	3 times < F ≤ 6 times	256 (6.3)	58.93 ± 17.08	
	>6 times	245 (6.0)	58.55 ± 16.40	
Daily sleep duration	≤ 6 h	963 (23.6)	58.50 ± 18.61	< 0.001
	6 h < T ≤ 7 h	2,205 (54.1)	65.02 ± 19.12	
	7 h < T ≤ 8 h	745 (18.3)	70.82 ± 21.01	
	>8 h	163 (4.0)	72.77 ± 25.06	
Weekly overtime hours	≤ 5 h	2,175 (53.4)	67.25 ± 20.64	< 0.001
	5 h < T ≤ 10 h	1,332 (32.7)	63.38 ± 18.89	
	10 h < T ≤ 15 h	317 (7.8)	60.80 ± 19.42	
	T>15 h	252 (6.2)	57.03 ± 18.78	
Average monthly earnings (RMB)	≤ 5000	1,182 (29.0)	62.79 ± 21.45	< 0.001
	5001-	1,927 (47.3)	65.01 ± 19.80	
	10001-	847 (20.8)	66.40 ± 18.82	
	≥20001	120 (2.9)	71.80 ± 16.73	
Hospital level	Primary hospital	231 (5.7)	58.37 ± 19.98	< 0.001
	Secondary hospital	1,462 (35.9)	64.16 ± 19.96	
	Tertiary hospital	2,383 (58.5)	65.91 ± 20.05	
Region	Pearl River Delta	2,426 (59.5)	63.72 ± 19.57	< 0.001
	Eastern	377 (9.2)	60.83 ± 19.98	
	Western	703 (17.2)	68.19 ± 22.28	
	Northern	570 (14.0)	68.19 ± 18.45	

The scores from each different dimension, self, organizational management, patient, social support, and environment, are shown in Table 2. Gender, work experience (years), education level, job position, specialty unit, employment type, marital status, job satisfaction, WPV frequency, daily sleep duration, weekly overtime hour, average monthly earnings (RMB), hospital level and region were all significantly correlated to job security (all *P* < 0.05) (Table 1). Between these variables, the tolerance limit was 0.1 or higher, ranging from 0.53 to 0.96. The variance inflation factor (VIF) ranged from 1.04 to 1.89, which was below the reference value of 3. Therefore, there was no multicollinearity among the independent variables. Multiple linear regression analysis showed that work experience (years), education level, job position, specialty unit, employment type, marital status, job satisfaction, WPV frequency, daily sleep duration, weekly overtime hours, average monthly earnings (RMB), hospital level, and region are significantly and independently associated with job security among healthcare workers (all *P* < 0.05) (Table 3).

**Table 2:** Job security scores across each dimension (*n* = 4076).

**Dimension**	**Score ranges**	**Total points**	**Points Normalized by Entries**	**Rank**	**Average Score, %**
Self	3–15	9.30 ± 3.13	3.10 ± 1.04	1	62.00
Organizational management	7–35	21.37 ± 7.41	3.05 ± 1.06	2	61.06
Patients	4–20	11.80 ± 4.45	2.95 ± 1.11	3	59.00
Social support	4–20	11.55 ± 5.60	2.75 ± 0.89	4	57.75
Environment	4–20	10.83 ± 4.02	2.71 ± 1.00	5	54.15
Total score	22–110	64.85 ± 20.09	2.95 ± 0.91		58.95

**Table 3:** Multiple linear regression analysis of influencing factors of healthcare workers security (*n* = 4076).

**Variables**	**Regression coefficient**	**95% *CI***	**Standard error**	**Standardized regression coefficient**	***P***
(Constant)	83.264	(78.781, 87.746)	2.286	–	< 0.001
**Gender**					
Male (ref)	–	–	–	–	–
Female	−0.382	(−1.666, 0.902)	0.655	−0.009	0.056
**Work experience (years)**					
≤ 5 (ref)	–	–	–	–	–
6–	−3.383	(−4.871, −4.871)	0.759	−0.976	< 0.001
11–	−6.121	(−7.875, −4.367)	0.895	−0.125	< 0.001
≥21	−7.762	(−9.827, −5.696)	1.954	−0.141	< 0.001
**Education level**					
Junior college or below (ref)	–	–	–	–	–
Bachelor's degree	−0.794	(−2.266, 0.678)	0.751	−0.019	0.029
Master's degree or above	4.779	(0.678, 7.876)	1.580	0.100	3.026
**Job position**					
Nurse (ref)	–	–	–	–	–
Doctor	−3.691	(−6.6, −0.783)	1.484	−0.080	0.013
Pharmacist	5.552	(2.665, 8.438)	1.472	0.086	< 0.001
Technologist	6.814	(8.438, 9.74)	1.493	0.099	< 0.001
**Specialty unit**					
**Outpatient/emergency (ref)**					
ICU	−5.180	(−8.111, −2.248)	1.495	−0.059	0.001
Medical	−0.974	(−3.027, −3.027)	1.047	−0.021	0.352
Surgical	−0.974	(−3.171, 1.222)	1.121	−0.018	0.385
Maternity	−1.828	(−4.789, 1.133)	1.510	−0.021	0.226
Pediatric	−3.510	(−7.011, –−0.008)	1.786	−0.031	0.049
Assistant department	−1.729	(−0.008, 1.010)	1.397	−0.038	0.216
Other	−3.270	(−6.546, 0.006)	1.671	−0.032	0.050
**Employment type**					
Permanent employee	–	–	–	–	–
Contract employee	−4.703	(−6.049, −3.357)	0.687	−0.116	< 0.001
Personnel agency employee	−4.189	(−8.415, 0.037)	2.156	−0.028	0.052
**Marital status**					
Married (ref)	–	–	–	–	–
Single	6.975	(5.717, 8.233)	0.642	0.165	< 0.001
Divorced/separated	−3.189	(−7.183, 0.805)	2.037	−0.022	0.118
**Job satisfaction**					
Yes (ref)	–	–	–	–	–
No	−9.322	(−10.648, −7.997)	0.676	−0.211	< 0.001
**WPV frequency**					
No (ref)	–	–	–	–	–
≤ 3 times	−6.120	(−7.33, −4.91)	0.617	−0.150	< 0.001
3 times < F ≤ 6 times	−7.497	(−9.884, −5.11)	1.218	−0.091	< 0.001
> 6 times	−8.550	(−11.005, −6.094)	1.252	−0.101	< 0.001
**Daily sleep duration**					
≤ 6 h (ref)	–	–	–	–	–
6 h < T ≤ 7 h	−13.388	(−16.371, −10.405)	1.522	−0.283	< 0.001
7 h < T ≤ 8 h	−9.187	(−12.054, −6.321)	1.462	−0.228	< 0.001
> 8 h	−5.375	(−8.429, −2.322)	1.557	−0.103	< 0.001
**Weekly overtime hour**					
≤ 5 h (ref)	–	–	–	–	–
5 h < T ≤ 10 h	−2.394	(−3.64, −3.64)	0.635	−0.056	< 0.001
10 h < T ≤ 15 h	−4.487	(−6.643, −2.330)	1.100	−0.060	< 0.001
T > 15 h	−7.429	(−9.851, −5.007)	1.235	−0.089	< 0.001
**Average monthly earnings (RMB)**					
≤ 5000 (ref)	–	–	–	–	–
5001–	0.141	(−1.316, 1.598)	0.743	0.004	0.849
10001–	0.185	(−1.829, 2.199)	1.027	0.004	0.857
≥ 20001	4.748	(0.955, 8.541)	1.935	0.040	0.014
**Hospital level**					
Primary hospital (ref)	–	–	–	–	–
Secondary hospital	4.908	(2.284, 7.532)	1.338	0.117	< 0.001
Tertiary hospital	5.074	(2.299, 7.849)	1.415	0.124	< 0.001
**Region**					
Pearl River Delta (ref)	–	–	–	–	–
Eastern	−2.888	(−4.968, −0.808)	1.061	−0.042	0.007
Western	0.615	(−1.099, 2.329)	0.874	0.012	0.482
Northern	0.779	(−0.973, 2.53)	0.893	0.013	0.384

## 4. Discussion

This study showed that these hospital workers perceived their workplace as relatively unsafe. Multiple-linear regression analysis showed that work experience (years), education level, job position, specialty unit, employment type, marital status, job satisfaction, WPV frequency, daily sleep duration, weekly overtime hours, average monthly earnings (RMB), hospital level, and region were significantly associated with feelings of poor safety and security among healthcare workers. This data may provide a reference for hospital managers to formulate policies that improve workplace safety.

Prior research demonstrates that effective team leadership is an essential factors for the team atmosphere (27). Therefore, the department directors and the head nurses can play a critical role in building feelings of job security among staff members.

Surprisingly, inexperience, as demonstrated by fewer years on the job, was associated with relatively high feelings of job security, a finding which was inconsistent with some reports (28). This discrepancy may be due to the relationship between age and career development. Older workers are more likely to have greater work experience and higher levels of education which can increase job security, but they are also more likely to experience sleep disturbances which negatively impact performance and job satisfaction. This is supported by the finding that sleep duration was inversely related to job security in this study.

In Guangdong, the State Council's regulation states the working week is 8 h a day for 40 h a week and any work exceeding these house is overtime work. In this study, over half of those surveyed worked 5 h of overtime a week. Previous studies showed that 61.2% of healthcare workers worked 5 h of overtime per week (29), while survey results of healthcare workers in Lanzhou show a rate more comparable to this study's 50% rate (30). According to the White Paper on the Occupational Status of Chinese doctors published in 2018, doctors in tertiary hospitals in China work an average of 51.5 h per week (5). Moreover, almost 60% of the healthcare workers in Ningbo Grade A hospitals work more than 8 h a day, 12.4% more than 10 h a day, and 4.8% more than 12 h a day (31). Overwork is more likely to cause errors or medical disputes, and thus weaken the sense of job security of healthcare workers (32).

Furthermore, this study found that employment at primary hospitals was associated with worse job security as compared with secondary and tertiary hospitals. In China, primary hospital are local community hospitals with fewer resources than the larger more specialized secondary and tertiary hospitals (33). The public and government officials generally equate patient volumes with the quality of care and as such large patient volumes have become a prerequisite to aquiring funding for high-quality training and research (34). Career development opportunities such as those offered by tertirary hospitals have been shown to be associated with greater levels of job security among hospital workers (35).

More than half of the healthcare workers in this survey experienced WPV during the yearlong study period. This suggests that medical departments need WPV training, and the hospital should strengthen personal safety protection measures among healthcare workers (36), especially in the department of surgery. Furthermore, the department in which a healthcare worker was employed was significantly associated with job security. This indicates that healthcare workers in high-risk departments, such as the emergency and critical care and obstetrics and gynecology departments, need more supports than other lower risk departments such as dermatology.

Additionally, home and environmental factors were shown to have a significant association with job security in this study. Married healthcare workers and those with higher incomes had greater levels job security on average than workers who were divorced or who had lower incomes. This confirms that marriage and family life can have a strong stabilizing influence on healthcare workers' lives and outlook (37). Since higher incomes are needed to support families, it follows that higher incomes were also associated with greater job security.

The survey results show that contract workers account for almost 6 in 10 workers, indicating that most of the healthcare workers in this survey belong to the contract system. Although the public hospitals claim to follow a policy of equal pay for equal work, contract staff have reduced benefits, fewer sick days, and higher stress (38). Hospitals in eastern Guangdong were ranked the lowest in this study, while those in northern Guangdong were ranked highest. These differences may be related to the economic development of each region, suggesting that hospital managers should adopt localized strategies.

This study had some limitations. This was a survey-based research study that captured the attitudes of medical workers toward their sense of job security in the workplace at a single point in time. As such, it is unknown if the trends observed in this study persist throughout the year or over time. Guangdong has a rapidly growing economy, and as such conditions may change rapidly that can improve the sense of job security among workers. Additional research should be carried out to understand the dynamic factors that influence the sense of job security among hospital workers and identify which strategies most improve the job security of healthcare workers.

## 5. Practice implication

Department managers should focus on the department construction. Efforts to build department unity and enhance collaboration may improve the mental health of healthcare workers and foster a sense of job security. Hosptials should equip key departments with one-button alarm devices and increase the intensity of security in high risk areas (39). Additionally, social support is key to mental health and worker wellbeing and should be a focus of hospital remediation efforts to mitigate the effects of unavoidable WPV (40).

## 6. Conclusions

In conclusion, the total job security score for healthcare workers in this study was relatively low. Education level, employment duration, job satisfaction, the frequency of WPV, daily sleep duration, overtime hours, professional category, departments, employment type, marital status, and locality of employment were significantly associated with feelings of poor safety and security among healthcare workers. A multi-center study with larger sample size is needed in future to make further conclusions about these results.

## Data availability statement

The original contributions presented in the study are included in the article/supplementary material, further inquiries can be directed to the corresponding author.

## Ethics statement

The studies involving human participants were reviewed and approved by the Guangdong Provincial People's Hospital Ethics Committee. The patients/participants provided their written informed consent to participate in this study. Written informed consent was obtained from the individual(s) for the publication of any potentially identifiable images or data included in this article.

## Author contributions

LM and HH conceived and designed the study. SW, QZ, ZB, and XY collected the data. WL did the statistical analysis and produced the tables and figures. QH wrote the initial draft. All authors subsequently critically edited the report, read, and approved the final edition.

## References

[1] Maslow AH. A theory of human motivation. Psychol Rev. (1943) 1:370–96. 10.1037/h0054346

[2] Bakr RH Jarrar MK Abumadini MS Al SA Larbi EB. Effect of leadership support, work conditions and job security on job satisfaction in a medical college. Saudi J Med Med Sci. (2019) 7:100–5. 10.4103/sjmms.sjmms_105_17 31080390 PMC6503694

[3] Deng J Guo Y Ma T Yang T Tian X. How job stress influences job performance among Chinese healthcare workers: a cross-sectional study. Environ Health Prev Med. (2019) 24:2. 10.1186/s12199-018-0758-4 30611191 PMC6320635

[4] Zhang X Li Y Yang C Jiang G. Trends in workplace violence involving health care professionals in china from 2000 to 2020: a review. Med Sci Monit. (2021) 27:e928393. 10.12659/MSM.928393 33417590 PMC7802374

[5] Chinese Medical Doctor Association. White Paper on Physician Practice in China. (2018). Available online at: http://www.cmda.net/rdxw2/11526.jhtml (accessed March 15, 2021).

[6] Sun S Wang W. Violence against Chinese health-care workers. Lancet. (2011) 9742:657. 10.1016/S0140-6736(11)60733-2 21601709

[7] Cheung JC Ho LT Cheng JV Cham E Lam KN. Staff safety during emergency airway management for COVID-19 in Hong Kong. Lancet Respir Med. (2020) 8:e19. 10.1016/S2213-2600(20)30084-9 32105633 PMC7128208

[8] Jing X. Analysis of the status quo of violence in the hospital outpatient workplace and its impact on the mental health of nurses. Manage Health Serv China. (2018) 3535:504–6.

[9] Rosenthal LJ Byerly A Taylor AD Martinovich Z. Impact and prevalence of physical and verbal violence toward healthcare workers. Psychosomatics. (2018) 59:584–90. 10.1016/j.psym.2018.04.007 29909013

[10] Wu LT Low MM Tan KK Lopez V Liaw SY. Why not nursing? A systematic review of factors influencing career choice among healthcare students. Int Nurs Rev. (2015) 62:547–62. 10.1111/inr.12220 26572517

[11] Xu Yan Y Yang L Zhang Y Yang X. Study on the relationship between job insecurity, work life quality and turnover intention of nurses. PLA Nursing J. (2017) 34:23–6.

[12] Biksegn A Kenfe T Matiwos S Eshetu G. Burnout status at work among health care professionals in a tertiary hospital. Ethiop J Health Sci. (2016) 26:101–8. 10.4314/ejhs.v26i2.3 27222622 PMC4864338

[13] Wang Jiao L Zhang Y Zeng L Wang Q Wang Y. Occupational stress and psychological security of clinical nurses in Changsha City and its correlation. Occupation and Health. (2016) 32:1623–8.

[14] Lo D Wu F Chan M Chu R Li D. A systematic review of burnout among doctors in China: a cultural perspective. Asia Pac Fam Med. (2018) 17:3. 10.1186/s12930-018-0040-3 29449785 PMC5806482

[15] ISBN. Framework Guidelines for Addressing Workplace Violence in the Health Sector. Geneva, Switzerland Ilo. (2002).

[16] Ramzi ZS Fatah PW Dalvandi A. Prevalence of workplace violence against healthcare workers during the COVID-19 pandemic: a systematic review and meta-analysis. Front Psychol. (2022) 13:896156. 10.3389/fpsyg.2022.896156 35712196 PMC9195416

[17] Liu J Gan Y Jiang H. Prevalence of workplace violence against healthcare workers: a systematic review and meta-analysis. Occup Environ Med. (2019) 76:927–37. 10.1136/oemed-2019-105849 31611310

[18] Li Z Yan CM Shi L Mu HT Li X Li AQ. Workplace violence against medical staff of Chinese children's hospitals: a cross-sectional study. PLoS ONE. (2017) 12:e179373. 10.1371/journal.pone.0179373 28609441 PMC5469493

[19] Zhao S Xie F Wang J Shi Y Zhang S Han X. Prevalence of workplace violence against chinese nurses and its association with mental health: a cross-sectional survey. Arch Psychiat Nurs. (2018) 32:242–7. 10.1016/j.apnu.2017.11.009 29579519

[20] Nyberg A Kecklund G Hanson LM Rajaleid K. Workplace violence and health in human service industries: a systematic review of prospective and longitudinal studies. Occup Environ Med. (2021) 78:69–81. 10.1136/oemed-2020-106450 32414952 PMC7873420

[21] Zhang L Wang A Xie X Zhou Y Li J Yang L. Workplace violence against nurses: a cross-sectional study. Int J Nurs Stud. (2017) 72:8–14. 10.1016/j.ijnurstu.2017.04.002 28412581

[22] Cheung T Yip PS. Workplace violence towards nurses in Hong Kong: prevalence and correlates. BMC Public Health. (2017) 17:196. 10.1186/s12889-017-4112-3 28196499 PMC5310001

[23] Pienaar J De Witte H Hellgren J Sverke M. The cognitive/affective distinction of job insecurity: validation and differential relations. Southern Afr Bus Rev. (2013) 17:1–22.

[24] Cong Zhong An Lijuan. Preliminary preparation and reliability and validity test of security scale. Chinese J Mental Health. (2004) 18: 97–9.

[25] Ba Zhiqiong H Zhan Q Yu X Li W Huang H. Establishment and reliability and validity test of medical staff Security scale Chinese. Occupational Med. (2021) 48:379–85.

[26] Kelley K Bilson DF Chattopadhyay B. Sequential accuracy in parameter estimation for population correlation coefficients. Psychol Methods. (2019) 24:492–515. 10.1037/met0000203 30829512

[27] Salas-Vallina A AJLÁ. The challenge of increasing employees' well-being and performance: how human resource management practices and engaging leadership work together toward reaching this goal. Hum Resour Manage-Us. (2021) 3:333–47. 10.1002/hrm.22021

[28] Yu X Zhao Y Li Y Hu C Xu H Zhao X. Factors associated with job satisfaction of frontline medical staff fighting against COVID-19: a Cross-Sectional study in china. Front Public Health. (2020) 8:426. 10.3389/fpubh.2020.00426 32850610 PMC7417651

[29] Lu Y Hu XM Huang XL Zhuang XD Guo P Feng LF. Job satisfaction and associated factors among healthcare staff: A cross-sectional study in Guangdong Province, China. BMJ Open. (2016) 6:e11388. 10.1136/bmjopen-2016-011388 27436667 PMC4964254

[30] Jiang L. Study on the correlation between shift and working hours and sleep quality of medical staff in Class A hospitals.[master's thesis]. Lanzhou: University of Lanzhou (2020).

[31] Zhou Jiangjin X Guo A. Analysis of chronic overwork of medical staff in Grade A hospitals hospital management forum. Nursing Open. (2013) 30:51–3.

[32] Gao Y Liu C Fan X Wu M Jiang Y. Issues related to the health status, work pressure and occupational environments of medical staff at level a tertiary public hospitals in Shanghai. Ann Palliat Med. (2021) 10:8203–14. 10.21037/apm-21-1777 34353103

[33] Dong S Millar R Shi C Dong M Xiao Y Shen J. Rating hospital performance in china: review of publicly available measures and development of a ranking system. J Med Internet Res. (2021) 23:e17095. 10.2196/17095 34137724 PMC8277410

[34] Pan J Qin X Hsieh CR. Is the pro-competition policy an effective solution for China's public hospital reform? Health Econ Policy Law. (2016) 11:337–57. 10.1017/S1744133116000220 27346712

[35] Falatah R Almuqati J Almuqati H Altunbakti K. Linking nurses' job security to job satisfaction and turnover intention during reform and privatization: a cross-sectional survey. J Nurs Manag. (2021) 29:1578–86. 10.1111/jonm.13279 33502052

[36] Civilotti C Berlanda S Iozzino L. Hospital-Based healthcare workers victims of workplace violence in Italy: a scoping review. Int J Environ Res Public Health. (2021) 18:5860. 10.3390/ijerph18115860 34072551 PMC8198045

[37] Zhao G Yin C. Impact of job control on hospital workers' safety performance: a moderated mediation analysis of the influences of hospital safety climate and social support. Nurs Open. (2022) 2:781–9. 10.1002/nop2.1345 36030533 PMC9834537

[38] Shang J You L Ma C Altares D Sloane DM Aiken LH. Nurse employment contracts in Chinese hospitals: impact of inequitable benefit structures on nurse and patient satisfaction. Hum Resour Health. (2014) 12:1. 10.1186/1478-4491-12-1 24418223 PMC3896777

[39] Qi M Hu X Liu J Wen J Hu X Wang Z. The impact of the COVID-19 pandemic on the prevalence and risk factors of workplace violence among healthcare workers in China. Front Public Health. (2022) 10:938423. 10.3389/fpubh.2022.938423 35958846 PMC9358256

[40] Sun L Zhang W Qi F Wang Y. Gender differences for the prevalence and risk factors of workplace violence among healthcare professionals in Shandong, China. Front Public Health. (2022) 10:873936. 10.3389/fpubh.2022.873936 35586009 PMC9108195

